# Traditional knowledge on health: balancing innovation, ethics and intellectual property

**DOI:** 10.2471/BLT.25.293487

**Published:** 2025-09-16

**Authors:** Sumeet Goel, Motlalepula Matsabisa, Fei Jiao, Caio Portella, Vijayalakshmi Asthana, Nilce Ekandzi, Manjeet S Saluja, Stefan Germann, João P Souza

**Affiliations:** a All India Institute of Ayurveda, Flat no. 3, Type 5 quarters Dhargal, Pernem, North Goa, 413513, India.; b African Medicines Innovations and Technologies Development, University of the Free State, Bloemfontein, South Africa.; c World Intellectual Property Organization, Geneva, Switzerland.; d Brazilian Academic Consortium for Integrative Health, São Paulo, Brazil.; e CSIR-Traditional Knowledge Digital Library Unit, New Delhi, India.; f Laboratoire du CEIPI, Strasbourg, France.; g Global Traditional Medicine Centre, World Health Organization, Jamnagar, India.; h Ursimone Wietlisbach Foundation, Zug, Switzerland.; i Latin American and Caribbean Center on Health Sciences Information, Pan American Health Organization/World Health Organization, Sao Paulo, Brazil.

## Abstract

Traditional knowledge on health has long contributed to global health-care systems. Rooted in the cultural and ecological heritage of Indigenous Peoples and local communities, traditional knowledge has influenced pharmaceutical research, biodiversity conservation and public health strategies. However, concerns over misappropriation of traditional knowledge and inadequate benefit-sharing with the sources of such knowledge persist. The World Intellectual Property Organization Treaty on Intellectual Property, Genetic Resources and Associated Traditional Knowledge mandates patent disclosure requirements for genetic resources and traditional knowledge. While a step forward, the treaty’s success depends on its effective implementation, ethical documentation of traditional knowledge, governance of artificial intelligence and equitable benefit-sharing mechanisms, among other factors. We examine traditional knowledge protection under intellectual property systems, the provisions of the World Intellectual Property Organization treaty, challenges to documentation of traditional knowledge and the role of artificial intelligence in the governance of traditional knowledge. By fostering a legally robust and technology-driven protection system for traditional knowledge, policy-makers can ensure that traditional knowledge remains both a protected cultural heritage and a resource for sustainable innovation in global health.

## Résumé

Les savoirs traditionnels en matière de santé contribuent depuis longtemps aux systèmes de santé mondiaux. Ancrées dans le patrimoine culturel et écologique des peuples autochtones et des populations locales, ces connaissances ont influencé la recherche pharmaceutique, la préservation de la biodiversité et les stratégies de santé publique. Toutefois, des préoccupations persistent quant à l’appropriation abusive des savoirs traditionnels et au partage inadéquat de leurs avantages avec les sources de ces connaissances. Le Traité de l’Organisation mondiale de la propriété intellectuelle (OMPI) sur la propriété intellectuelle, les ressources génétiques et les savoirs traditionnels associés établit une exigence de divulgation dans les demandes de brevet pour les ressources génétiques et les savoirs traditionnels. Bien qu’il s’agisse d’un progrès certain, le succès de ce traité dépend, entre autres facteurs, de sa mise en œuvre effective, de la documentation éthique des savoirs traditionnels, de la gouvernance de l’intelligence artificielle et des mécanismes équitables de partage des avantages liés. Nous examinons la protection des savoirs traditionnels dans le cadre des systèmes de propriété intellectuelle, des dispositions du traité de l’OMPI, des défis liés à la documentation des savoirs traditionnels et du rôle de l’intelligence artificielle dans la gouvernance de ces connaissances. En favorisant un système de protection solide sur le plan juridique et axé sur la technologie, les décideurs politiques seront capables de garantir la préservation des savoirs traditionnels en tant que patrimoine culturel et ressource pour l’innovation durable dans le domaine de la santé mondiale.

## Resumen

Los conocimientos tradicionales sobre salud han contribuido desde hace tiempo a los sistemas de atención sanitaria a nivel mundial. Arraigados en el patrimonio cultural y ecológico de los pueblos indígenas y las comunidades locales, los conocimientos tradicionales han influido en la investigación farmacéutica, la conservación de la biodiversidad y las estrategias de salud pública. Sin embargo, persisten las preocupaciones por la apropiación indebida de los conocimientos tradicionales y la distribución inadecuada de beneficios con las fuentes de dichos conocimientos. El Tratado de la Organización Mundial de la Propiedad Intelectual sobre Propiedad Intelectual, Recursos Genéticos y Conocimientos Tradicionales Asociados establece requisitos de divulgación de patentes para los recursos genéticos y los conocimientos tradicionales. Aunque representa un avance, el éxito del tratado depende de su aplicación efectiva, la documentación ética de los conocimientos tradicionales, la gobernanza de la inteligencia artificial y mecanismos equitativos de distribución de beneficios, entre otros factores. Se examina la protección de los conocimientos tradicionales en los sistemas de propiedad intelectual, las disposiciones del tratado de la Organización Mundial de la Propiedad Intelectual, los desafíos para la documentación de los conocimientos tradicionales y el papel de la inteligencia artificial en su gobernanza. Al fomentar un sistema de protección jurídicamente sólido y basado en la tecnología para los conocimientos tradicionales, los responsables de la formulación de políticas pueden garantizar que los conocimientos tradicionales sigan siendo tanto un patrimonio cultural protegido como un recurso para la innovación sostenible en la salud mundial.

## ملخص

لقد أسهمت المعارف التقليدية في مجال الصحة كثيرًا في تطوير أنظمة الرعاية الصحية العالمية. ولأن جذورها متأصلة في التراث الثقافي والبيئي للشعوب الأصلية والمجتمعات المحلية، فقد أثرت المعارف التقليدية على الأبحاث الدوائية، وحفظ التنوع البيولوجي، واستراتيجيات الصحة العامة. ومع ذلك، لا تزال هناك مخاوف بشأن سوء استخدام المعارف التقليدية وعدم كفاية مشاركة المنافع مع مصادرها. إن معاهدة المنظمة العالمية للملكية الفكرية بشأن الملكية الفكرية، والموارد الجينية، والمعارف التقليدية المرتبطة، تنص على متطلبات الإفصاح عن براءات الاختراع المتعلقة بالموارد الجينية والمعارف التقليدية. ورغم أن هذه المعاهدة تُعدّ خطوةً للأمام، إلا أن نجاحها يعتمد على تنفيذها الفعال، والتوثيق الأخلاقي للمعارف التقليدية، وحوكمة الذكاء الاصطناعي، وآليات مشاركة المنافع بشكل عادل، فضلاً عن عوامل أخرى. نحن نتحقق من حماية المعارف التقليدية في ظل أنظمة الملكية الفكرية، وأحكام معاهدة المنظمة العالمية للملكية الفكرية، والتحديات التي تواجه توثيق المعارف التقليدية، ودور الذكاء الاصطناعي في حوكمة المعارف التقليدية. ومن خلال تعزيز نظام حماية قوي من الناحية القانونية ومدفوع بالتكنولوجيا للمعرفة التقليدية، يستطيع صناع السياسات ضمان بقاء المعرفة التقليدية تراثاً ثقافياً محمياً، ومورداً للابتكار المستدام في مجال الصحة العالمية.

## 摘要

长期以来，卫生相关传统知识在促进全球卫生保健体系发展方面一直发挥着积极作用。根植于土著人民和地方社区的文化和生态遗产中的传统知识始终影响着药物研究和生物多样性养护的开展以及公共卫生战略的制定。然而，盗用传统知识以及未能与此类传统知识来源方适当进行惠益分享的问题持续存在。世界知识产权组织的《知识产权、遗传资源和相关传统知识条约》中规定了关于遗传资源和传统知识的专利披露要求。尽管该条约的签署是一个重要的里程碑，但是对于该条约能否成功发挥作用，还取决于其有效实施、传统知识的伦理文件编制、人工智能治理、公平的惠益分享机制以及其他因素。我们探讨了知识产权体系项下的传统知识保护问题、世界知识产权组织条约中的相关规定、在传统知识文件编制方面面临的挑战以及人工智能在传统知识治理方面所发挥的作用。政策制定者构建了一个在法律上比较健全的技术驱动型传统知识保护体系，从而能够确保传统知识不仅是一种受保护的文化遗产，还是全球卫生领域的一种可持续创新资源。

## Резюме

Традиционные медицинские знания долгое время вносили вклад в мировую систему здравоохранения. Традиционные знания, уходящие корнями в культурное и экологическое наследие коренного населения и местных общин, оказывали влияние на фармацевтические исследования, сохранение биологического разнообразия и стратегии общественного здравоохранения. Однако сохраняется озабоченность незаконным присвоением традиционных знаний и неравным распределением благ в отношении источников такого знания. Договор Всемирной организации интеллектуальной собственности по вопросам интеллектуальной собственности, генетических ресурсов и связанного с ними традиционного знания устанавливает требования к раскрытию для пациентов информации о генетических ресурсах и традиционных знаниях. Хотя это является шагом вперед, успех договора зависит среди прочего от его эффективного внедрения, этичного документирования традиционных знаний, регулирования искусственного интеллекта и механизма равного распределения благ. Авторы изучили вопросы защиты традиционных знаний в рамках существующих систем интеллектуальной собственности, положения договора Всемирной организации интеллектуальной собственности, проблемы с документированием традиционных знаний и роль искусственного интеллекта в регулировании традиционных знаний. Содействуя созданию надежной с правовой точки зрения и технологичной системы защиты традиционных знаний, лица, определяющие политики, могут гарантировать, что традиционные знания останутся в качестве как охраняемого культурного наследия, так и ресурса для устойчивых инноваций в сфере мирового здравоохранения.

## Introduction

Traditional knowledge on health has long contributed to global health-care systems. Rooted in the cultural and ecological heritage of Indigenous Peoples and local communities, traditional knowledge has influenced pharmaceutical research, biodiversity conservation and public health strategies.^1^^,^^2^

While few truly successful models exist in which people with traditional knowledge actively participate in research, innovation and commercialization, such as negotiated community agreements in Kenya, concerns about misappropriation of traditional knowledge and inadequate benefit-sharing continue.^3^

Historical colonial extraction, that is, systematic appropriation of medicinal plants and traditional knowledge without recognition or fair return (for example, cinchona and rubber), underlies present-day cases of biopiracy involving turmeric, neem and hoodia, called biocolonialism.^4^^,^^5^ Numerous cases of biopiracy have emerged, where traditional remedies and genetic resources have been commercialized without free, prior and informed consent of the knowledge holders or fair benefit-sharing arrangements. These cases highlight the need for stronger legal safeguards and enforcement measures.^6^^,^^7^ Strengthening national and international frameworks and promoting fair collaboration models can safeguard traditional knowledge and create opportunities for inclusive and responsible innovation. The emergence of advanced technologies, including artificial intelligence and biotechnology,^8^^,^^9^ has amplified the risk of biopiracy, particularly through practices such as digital sequencing, information-mining and bioinnovation driven by artificial intelligence.^10^^,^^11^ These developments further complicate protection of traditional knowledge and create new ethical and legal challenges for the governance of intellectual property.

The 2024 World Intellectual Property Organization (WIPO) Treaty on Intellectual Property, Genetic Resources and Associated Traditional Knowledge is a major milestone in addressing these issues.^12^ The treaty has introduced a mandatory patent disclosure requirement in relation to genetic resources and traditional knowledge associated with genetic resources. This mandate requires patent applicants to reveal the country of origin of the genetic resources and/or the Indigenous Peoples and local communities providing the traditional knowledge associated with genetic resources, if the claimed inventions are based on such resources and/or knowledge.

While the treaty represents progress in improving the transparency of patent systems, its effective implementation requires context-specific strategies and several challenges remain. Enforcing disclosure requirements, strengthening national patent office capacities and closing regulatory loopholes require comprehensive national and international strategies.^13^ The documentation of traditional knowledge, especially oral and sacred knowledge, raises concerns about accessibility, cultural sensitivity, cybersecurity, ethical data governance, control and ownership over traditional knowledge and the data extracted from traditional knowledge and genetic resources. Additionally, artificial intelligence (AI) can analyse repositories of traditional knowledge and digital sequence information on genetic resources and generate patentable innovations without engaging traditional knowledge holders. This capability could result in automated biopiracy, where traditional knowledge is systematically mined and commercialized without free, prior and informed consent.^10^^,^^11^^,^^14^

Various international organizations, including the World Health Organization (WHO) and WIPO, continue to explore ethical and legal frameworks for protection of traditional knowledge. The WIPO Intergovernmental Committee on Intellectual Property and Genetic Resources, Traditional Knowledge and Folklore is still engaged in text-based negotiations on draft international legal instruments concerning traditional knowledge and traditional cultural expression.^15^ A key development in this effort was the global technical meeting convened by the WHO Global Traditional Medicine Centre and the Department of Digital Health and Innovation in September 2024 at the All-India Institute of Ayurveda in New Delhi, India.^16^ This meeting focused on the intersection of AI, intellectual property and traditional medicine, and underscored the urgent need for harmonized international policies. Key issues discussed included the development of AI governance frameworks tailored to traditional medicine; the establishment of secure and interoperable traditional knowledge databases; and the ethical integration of traditional knowledge into health-care systems, with a strong emphasis on upholding the rights of Indigenous Peoples. A persistent challenge is balancing technological innovation and economic interests with the rights of Indigenous Peoples and local communities. Addressing this issue requires a globally coordinated intellectual property framework that recognizes traditional knowledge as both a cultural heritage and a scientifically valuable asset, and ensures equitable benefit-sharing and responsible use in a health-care landscape that is driven by AI.^16^^,^^17^

## Intellectual property protection 

Conventional intellectual property systems can play an important role in protecting traditional knowledge, either positively (i.e. granting intellectual property rights over the subject matter of traditional knowledge) or defensively (i.e. preventing third parties from claiming intellectual property rights over traditional knowledge). In many countries, pharmaceutical products based on traditional knowledge can be protected with patents as long as such products are novel (i.e. not previously known, published or disclosed anywhere in the world), inventive (i.e. not an obvious or trivial modification of existing knowledge to a person skilled in the field) and industrially applicable. The patent systems can also be used to protect traditional knowledge defensively by preventing patents from being granted improperly. Examples of tackling patents that have been erroneously granted include revoking a patent on the use of turmeric for healing wounds, and a patent on the antifungal properties of neem.^18^

However, gaps exist in the conventional intellectual property systems in relation to protection of traditional knowledge. For example, a traditional medicine that has been used for generations cannot be protected by patents due to lack of novelty. Some countries (e.g. Kenya and Thailand) and a regional organization (African Regional Intellectual Property Organization, via the 2010 Swakopmund Protocol) have adopted *sui generis* frameworks for traditional knowledge, that is, specialized legal systems tailored to protecting traditional knowledge beyond conventional intellectual property laws.^4^

## The treaty

The WIPO treaty^12^ builds on decades of negotiations within WIPO’s Intergovernmental Committee on Intellectual Property and Genetic Resources and Associated Traditional Knowledge.^15^ Adopted in 2024 after nearly 25 years of negotiation, the treaty’s practical impact now depends on broad ratification, including by high-income jurisdictions, to avoid uneven application of disclosure obligations and uncertainty where traditional knowledge or associated genetic resources have multiple national sources.^12^^,^^14^^,^^19^ The treaty represents a diplomatic convergence between biodiversity-rich developing nations advocating for stringent anti-biopiracy measures and industrialized countries seeking legal clarity for patent applicants.^20^

A key provision of the treaty is the mandatory disclosure for patents based on genetic resources and traditional knowledge associated with genetic resources. Patent applicants must disclose the country of origin of the genetic resources and/or the Indigenous Peoples or local communities providing the traditional knowledge, if the claimed inventions are based on these resources. If such information is unknown, the source should be disclosed. The source of genetic resources among others includes research centres, gene banks, and Indigenous Peoples and local communities; while the source of traditional knowledge associated with genetic resources includes scientific literature, publicly accessible databases, patent applications and patent publication.

By disclosing such information, the treaty enhances the effectiveness, transparency and quality of the patent system with regard to genetic resources and traditional knowledge associated with genetic resources. The treaty also prevents patents from being granted erroneously for inventions that are not novel or inventive as they are based on existing genetic resources or on traditional knowledge linked to those resources. Subject to existing national laws, no obligations of the treaty should be imposed on patent applications filed before the entry into force of the treaty.^12^

Although the treaty does not directly address access and benefit-sharing, it could potentially bridge the gaps between intellectual property and access and benefit-sharing mechanisms such as those established by the Convention on Biological Diversity and its Nagoya Protocol on access and benefit-sharing.^21^^,^^22^ The treaty could make it easier for the countries of origin and Indigenous Peoples and local communities to share in the benefits that arise from the use of their genetic resources and traditional knowledge associated with genetic resources.^19^

The effectiveness of the treaty’s requirements hinges on national implementation and enforcement mechanisms. Many national patent offices lack the expertise and resources to verify disclosure statements or assess compliance with access and benefit-sharing laws.^14^ The treaty also specifies that patent offices are not obligated to verify the authenticity of these disclosures. Additionally, patents cannot be revoked solely for non-disclosure unless fraudulent intent is proven. Furthermore, the treaty allows national authorities significant flexibility in implementing it.^19^ The treaty includes a reference to the establishment of databases of genetic resources and traditional knowledge associated with genetic resources to support patent search and examination. Where applicable, these databases should be developed in consultation with Indigenous Peoples and local communities, but the practical implementation is vague.^23^ Notably, Article 4 introduces a partial retroactive element applying to genetic resources or traditional knowledge accessed by patent applicants before the treaty’s entry into force, which could have considerable implications for the pharmaceutical sector and ongoing research involving past access.^12^

Because the treaty will enter into force only after 15 ratifications and it is not yet universal, its disclosure requirements may be circumvented in non-party jurisdictions, particularly where traditional knowledge spans multiple countries.^12^^,^^20^ A harmonized access and benefit-sharing structure under the Convention on Biological Diversity^21^ and Nagoya Protocol^22^ therefore remains essential. Together, the Convention on Biological Diversity and Nagoya Protocol, the treaty and the newly adopted WHO Pandemic Agreement^24^ with its pathogen access and benefit-sharing annex create an interlinked international system which shapes governance of genetic resources and associated traditional knowledge in both biodiversity and public health emergencies. The rapid advancement of AI intensifies the challenges by enabling large-scale extraction and use of genetic and traditional knowledge data, which raises new risks of biopiracy driven by AI unless global governance frameworks adapt accordingly.^25^^,^^26^

## Documentation and governance

The documentation of traditional knowledge, particularly oral traditions and sacred knowledge, presents significant policy and ethical dilemmas. Unlike scientific knowledge, traditional knowledge is often transmitted orally across generations, making it difficult to establish prior art (i.e. evidence that an invention or knowledge existed before a patent application was filed) in patent disputes.^4^ Moreover, certain forms of traditional knowledge, such as healing rituals, sacred plant knowledge or ceremonial practices, are culturally sensitive and not intended for public disclosure. Balancing the need for defensive documentation and positive protection while respecting the rights and dignity of Indigenous Peoples and local communities remains a challenge.^27^

Several countries have adopted proactive approaches to document and protect traditional knowledge. India’s Traditional Knowledge Digital Library compiles knowledge from ayurveda, unani, siddha, sowa rigpa and yoga systems, making it accessible to patent offices while preventing the erroneous granting of patents.^28^ China’s traditional Chinese medicine database integrates pharmacopeial data into patent examination processes, ensuring traditional knowledge is respected when assessing innovation claims. Brazil’s access and benefit-sharing database mandates disclosure of traditional knowledge use in research and commercialization, and aligns protection of traditional knowledge with policies on biodiversity conservation.^29^

In parallel, community-led documentation, such as digital archives in Assam, India, showcases locally driven efforts that respect cultural values and community agency. However, codifying oral and sacred traditions into formal systems poses risks of oversimplification, cultural misrepresentation and potential violation of moral rights.^30^

Documentation of traditional knowledge is inherently complex as it encompasses cultural, sacred and ritualistic dimensions. Ethical concerns about recording and sharing culturally sensitive information must be prioritized to prevent misappropriation or commodification.

Governance of access to traditional knowledge databases adds further complexity. Policy-makers face difficult questions, such as whether access should be universal for all patent offices or selectively restricted to prevent misuse. Establishing a clear framework to differentiate knowledge that can be publicly shared from oral, sacred or secret knowledge is essential to uphold *sui generis* rights, protect cultural integrity and maintain community control. India’s Traditional Knowledge Digital Library demonstrates how restricted sharing with patent offices deters biopiracy without making formulations public.^28^ Blockchain tools can further secure provenance, embed community consent and enhance traceability.^31^ Additionally, the principles of Indigenous data sovereignty underscore the need for community-governed repositories and access protocols based on free, prior and informed consent.^32^ Together, these measures provide a culturally respectful, rights-based path for safeguarding traditional knowledge while enabling transparent, innovation-friendly and prior-art verification.

Equally important is the need for capacity-building initiatives that empower Indigenous Peoples and local communities to actively participate in documenting, monitoring and governing access to their knowledge. Such capacity-building aligns with the treaty’s explicit acknowledgement of the United Nations Declaration on the Rights of Indigenous Peoples and its commitment to realizing the principles of the Declaration.^23^ WIPO supports these efforts through training programmes,^33^ while organizations such as WHO can help develop ethical frameworks and provide technical support to countries that possess an abundance of traditional knowledge.

## Artificial intelligence

### Traditional knowledge documentation

AI can analyse large data sets, identify patterns, detect potential biopiracy and help translate ancient texts. Such applications can make traditional knowledge more accessible, but it also poses serious ethical concerns if used without the free, prior and informed consent of Indigenous Peoples and local communities.^17^ The risks include data privacy, ownership and potential misappropriation through cyber breaches or automated extraction.^34^

To address these risks, governments could require transparency on AI training data and develop traditional knowledge databases under the oversight of Indigenous Peoples and local communities. Blockchain technology can support these efforts by creating secure, traceable ownership records tied to free, prior and informed consent and benefit-sharing agreements. By integrating strong governance frameworks, AI can help protect traditional knowledge at an affordable cost while respecting the rights and legitimate aspirations of Indigenous Peoples and local communities and cultural integrity.

### Traditional knowledge protection

Integrating AI in biotechnology and life sciences offers opportunities but also risks for protecting traditional knowledge. AI tools can analyse traditional knowledge repositories, identify bioactive compounds and generate novel drug formulations, which can potentially lead to patent claims without the free, prior and informed consent of Indigenous Peoples and local communities.

While the treaty does not explicitly regulate AI applications in traditional knowledge, Article 8 allows for future reviews and possible extensions of disclosure requirements to new technologies, including AI, four years after the treaty’s entry into force.

Given AI’s growing role in bioinformatics, national policies could require companies to disclose their training data sources and obtain explicit free, prior and informed consent before commercialization. Additionally, patent examiners could use AI tools to detect automated biopiracy, thus helping to prevent unauthorized patents and ensuring benefit-sharing agreements are upheld.^14^

Recognizing the need for clear regulation of AI in traditional medicine, WHO and the International Telecommunication Union launched the Topic Group on AI for Traditional Medicine under the Focus Group on AI for Health in 2022.^35^ This initiative aims to set quality benchmarks for AI assessments of traditional medicine; develop tailored AI systems; and promote responsible use of AI in research and practice. These efforts highlight the importance of ethical and transparent governance of AI in this field.

Additionally, national guidelines are needed on the use of AI in traditional knowledge research and documentation. Governments and policy-makers could develop ethical AI frameworks in collaboration with Indigenous Peoples and local communities to ensure traditional knowledge is protected and responsibly integrated into innovation driven by AI.

## Policy strategies

Comprehensive national policies are required to balance protection of traditional knowledge with innovation incentives. Governments and policy-makers could consider the following strategies.

### Traditional knowledge documentation

National governments should invest in secure, community-driven traditional knowledge documentation systems, developed and governed in close consultation with Indigenous Peoples and local communities. By creating national databases of traditional knowledge with restricted access, sensitive knowledge can be protected while equipping patent examiners with tools to assess prior art. Blockchain technology can further enhance security through transparent and tamper-proof ownership records. Legal frameworks for these databases could adopt either a defensive or positive approach, depending on national or regional strategies. Such frameworks should consider digital sequence information on genetic resources under the Nagoya Protocol, and the access and benefit-sharing multilateral mechanism and global fund adopted at the 2024 Convention on Biological Diversity Conference^36^

Additionally, to ensure ethical and equitable data stewardship, national and international information systems on traditional knowledge and genetic resources can incorporate data governance principles, including the principles of findability, accessibility, interoperability and reusability; and collective benefit, authority to control, responsibility and ethics.

### Patent examination

Patent examination processes need to be strengthened through capacity-building initiatives. Training patent examiners on the nuances involved, including the details and differences specific to traditional knowledge-based applications and their assessment, is important for effective enforcement of the requirements of patent disclosure. Verification of disclosure claims would put further pressure on intellectual property offices. However, with adequate investment in examiner training, better information-sharing systems and possibly AI tools, patent offices would be able to conduct these tasks; indeed, many are already doing so (e.g. the Indian patent office). While integration of AI for prior-art searches could be part of the solution, it requires careful consideration beforehand.

### Regulations

Appropriate regulations on the use of AI need careful integration into frameworks for the protection of traditional knowledge. Governments could require companies to disclose whether their AI models have been trained on traditional knowledge sources. In collaboration with Indigenous Peoples and local communities, ethical AI principles can be developed to safeguard Indigenous rights and prevent algorithmic exploitation of traditional knowledge.

AI systems also need to adhere strictly to human rights standards to ensure that they do not reinforce discrimination, perpetuate historical injustices or enable the commercialization of traditional knowledge without consent.

An AI framework compliant with human rights would ensure that applications respect free, prior and informed consent principles as outlined in the United Nations Declaration on the Rights of Indigenous Peoples. This safeguard is particularly important in traditional medicine, where models driven by AI may extract, classify or even alter the knowledge of Indigenous Peoples and local communities for commercial use without the explicit permission of the knowledge holders. Without robust human rights and traditional knowledge *sui generis* protections, AI could facilitate biopiracy, erode cultural autonomy and undermine the governance that Indigenous Peoples and local communities have over their own medicinal systems.

Furthermore, AI models trained on traditional knowledge need to be transparent and accountable to the communities from which the knowledge originates. The Nagoya Protocol highlights the importance of fair and equitable access and benefit-sharing when genetic resources and associated traditional knowledge are used. AI systems processing traditional knowledge need to align with such frameworks to prevent the unjust, unethical and increasingly unlawful appropriation of the knowledge held by Indigenous Peoples and local communities.

Ensuring that AI is both ethical and compliant with human rights is essential to fostering trust between AI developers and Indigenous Peoples and local communities, preventing the reinforcement of colonial or neocolonial extractive practices, and maintaining traditional knowledge under the stewardship of its rightful holders. Adapting data privacy principles, for example, privacy by design and privacy by default, to the context of protecting traditional knowledge and medicine and the rights of Indigenous Peoples and local communities, together with frameworks such as the CARE Principles for Indigenous Data Governance (collective benefit, authority to control, responsibility and ethics), could help build responsible and ethical AI.^32^ Without these safeguards, AI could become a tool of exploitation rather than empowerment in traditional medicine.^32^^,^^37^

### International cooperation

International cooperation is essential for bringing together national intellectual property policies and global frameworks for the protection of traditional knowledge. Aligning national laws with WIPO disclosure requirements, integrating traditional knowledge protection into biodiversity and health regulations, and advocating for stronger benefit-sharing mechanisms will create a more cohesive global approach to the governance of traditional knowledge.^20^

By strengthening multilateral cooperation, aligning international legal frameworks and using digital technologies responsibly, **governments and policy-makers** can create a more equitable and transparent system for protection of traditional knowledge. Such a system can advance the aims of the United Nations Declaration on the Rights of Indigenous Peoples while fostering responsible innovation in traditional medicine.

## Conclusion

Protecting traditional knowledge requires inclusive documentation, strengthened patent examination, ethical AI governance and strong international cooperation. By integrating equity, transparency, and free, prior and informed consent principles, **governments and policy-makers** can safeguard traditional knowledge, support equitable benefit-sharing and enable responsible innovation in traditional medicine. Combining legal safeguards with emerging technologies will help ensure that Indigenous Peoples and local communities retain control over their knowledge while preventing misappropriation and fostering culturally respectful use.

The treaty is a significant step forward in safeguarding traditional knowledge and preventing biopiracy.^13^ However, its success depends on robust national implementation and policy convergence across jurisdictions. As Indigenous leaders noted in a joint statement at the Diplomatic Conference on the treaty, “Our knowledge frequently lies at the heart of innovative discoveries and it has been exploited for centuries. This treaty is a first step towards guaranteeing just and transparent access to these resources.”^38^

Governments are encouraged to develop secure databases of traditional knowledge and include Indigenous Peoples and local communities in every step of the design, governance and implementation. Such action will enhance patent examination capabilities and regulate traditional knowledge research driven by AI. By fostering a protection system for traditional knowledge that is legally robust and technology-driven (defensive or positive), policy-makers can ensure that traditional knowledge remains both a protected and proactively conserved cultural heritage and a resource for sustainable innovation in the global bioeconomy.
